# Evaluation of Dietary Organic and Inorganic Mercury Threshold Levels on Induced Mercury Toxicity in a Marine Fish Model

**DOI:** 10.3390/ani10030405

**Published:** 2020-02-29

**Authors:** Said Majdood Raihan, Mohammad Moniruzzaman, Youngjin Park, Seunghan Lee, Sungchul C. Bai

**Affiliations:** 1 Department of Marine Bio-Materials and Aquaculture/Feeds & Foods Nutrition Research Center (FFNRC), Pukyong National University, Busan 608-737, Korea; rahan_1988@yahoo.com (S.M.R.); mzaman_bfri@yahoo.com (M.M.); shlee5863@naver.com (S.L.); 2 Jeju International Animal Research Center (JIA), Jeju National University, Jeju 63243, Korea; 3 Faculty of Biosciences and Aquaculture, Nord University, Universitetsallen 11, 8049 Bodø, Norway; xxmain@naver.com

**Keywords:** organic mercury, inorganic mercury, induced toxicity, broken-line model, olive flounder

## Abstract

**Simple Summary:**

This investigation was executed to establish the threshold level of inorganic and organic mercury incorporated in the diet of juvenile olive flounder in relation to the broken-line regression model for the percentage of weight gain of fish. Organic mercury incorporated diet resulted in more toxic behavior than its counterpart inorganic mercury in olive flounder. Mercury was found to be more biomagnified in kidney tissue than liver and gill tissues of fish. The study has importance in terms of knowledge on mercury toxicity in marine fish.

**Abstract:**

Mercury as one of the most toxic elements can be present in organic or inorganic form in marine fishes, which may cause a potential threat to public health. In this study, we investigated to determine the dietary organic (O-Hg) and inorganic (I-Hg) mercury threshold levels on induced mercury toxicity in juvenile olive flounder, *Paralichthys olivaceus* as a marine fish model. Twenty-eight fish averaging 3.1 ± 0.05 g (mean ± SD) were arbitrarily assigned to each of 27 tanks. Each tank was arbitrarily restricted to triplicates of nine experimental diets for eight weeks. The experimental diets were manufactured to contain 0 (Control), 10 (I-Hg10, O-Hg10), 20 (I-Hg20, O-Hg20), 40 (I-Hg40, O-Hg40) and 160 (I-Hg160, O-Hg160) mg/kg diet in organic form as methylmercury (MeHg) or in inorganic form as mercuric chloride (HgCl_2_). At the termination of the experimental trial, weight gains (WGs) of fish fed the control and 10 (I-Hg10, O-Hg10) diets were remarkably higher than those of fish fed the 20 (I-Hg20, O-Hg20), 40 (I-Hg40, O-Hg40) and 160 (I-Hg160, O-Hg160) (*p* < 0.05). Specific growth rate and feed efficiency of fish fed control and 10 (I-Hg10, O-Hg10) diets were significantly higher than those of fish fed 40 (I-Hg40, O-Hg40) and 160 (I-Hg160, O-Hg160) diets. In comparison to the dietary inorganic mercury, dietary MeHg bioaccumulation rates were significantly higher in the tissue levels according to the dietary inclusion levels. MeHg accumulated mostly in kidney, followed by liver and gill tissues. HgCl_2_ accumulated in tissues, in decreasing order, liver > kidney > gills. A broken-line regression model for percentage of WG indicated that the threshold toxicity level for an Hg-incorporated diet of juvenile olive flounder could be 13.5 mg Hg/kg in the form of HgCl_2_ and 8.7 mg Hg/kg in the form of MeHg.

## 1. Introduction

Mercury (Hg) is a naturally occurring element found in air, water, and soil [1,2]. It may exist in three chemical forms: (1) natural mercury, which may be found in liquid and gaseous states; (2) inorganic mercury compounds, including mercurous chloride (Hg_2_Cl_2_), mercuric chloride (HgCl_2_) and mercuric sulfide (HgS); and (3) organic mercury compound, especially methylmercury (MeHg) [3]. All forms of mercury are toxic [4], and organic MeHg is neurotoxic compared to inorganic HgCl_2_ in fish [5]. Although mercury is a naturally occurring element, human or anthropogenic activities, particularly through industrial processes, have led to widespread distribution of toxic mercurial derivatives throughout the biosphere [6]. In aquatic conditions, the natural mercury is biologically transformed into organic mercury by different types of microorganisms, basically anaerobic bacteria [7]. Mercury concentrations in fish vary with tissue type, species, age, sex, biometrics (weight and length), temperature and trophic level, as well as the location of fish [8,9,10]. The typical concentration of Hg in edible tissues of various species of fish ranges from 50 to 1400 mg/kg of fresh weight; however, fish from contaminated aquatic environments can have 10 mg/kg [11]. In a recent study in the Republic of Korea, the total mercury concentrations tended to be higher in predatory fish species such as sharks, billfishes, and tuna [12]. Niimi and Kissoon [13] reported that the level could reach 20 mg/kg, which may cause toxicity in fish. 

Mercury is considered a dangerous contaminant that can persist in water and sediment, cause high toxicity to living organisms, as well as bioaccumulate and in turn be biomagnified in the total food chain [14,15,16]. Soil with a low pH and high level of mercury can alter the methylmercury content of the aquatic system [7]. The acidified lakes can increase the methylation of mercury, as well as also decrease the diversity of fish in the aquatic environment by resulting bioaccumulation of mercury in their body. Anaerobic conditions [17] and increasing dissolved carbon levels [7] both can exclusively increase the methylation process of mercury. In this regard, fish are widely used as a potential organism in aquatic toxicology study to understand the environmental condition [14]. In addition, fish, especially marine fish, have a great ability to metabolize, accumulate and concentrate environmental pollutants such as mercury [15]. However, bioaccumulation of Hg in marine fish is highly variable, which may affect the survival, growth, and behavior of fish based on Hg accumulation patterns [18]. 

The transformation process of inorganic to organic mercury is very important in terms of: (1) higher toxicity of organic mercury than inorganic mercury, and (2) prolonged time of elimination of organic mercury by the animals, compared to inorganic mercury [1]. In light of this view, organic mercury bearing microorganisms ingested by the higher animals, or the microorganisms freeing the organic mercury to the aquatic environment may be rapidly absorbed by the plankton that may also be ingested by the next level of organism in the food-web [19,20].

The central nervous system is a critical target for Hg toxicity in all living organisms [8]. Inorganic and organic mercury are both responsible for damaging the brain of bony fish [20]. The methylmercury (organic form of mercury) can rapidly move to the central nervous system, which may cause more toxic effects than the inorganic mercury [20,21]. Pereira et al. [22] reported that a high level of MeHg can be found in the brain, eye wall and lens of golden grey mullet fish. However, for the first time, Cardoso et al. [23] postulated that inorganic mercury (Hg_2_Cl_2_) showed a high neurotoxicity potential in terms of the poor antioxidant system of the brain and recurrent oxidative damage, in comparison to MeHg exposure in fish. Methylmercury can be comfortably biomagnified in the food-web by the ingestion of fish in the aquatic environment [24]. Inorganic mercury can be present in the intestinal tissue of fish after methylation process [20,25], or it can be directly taken up, which may create a more toxic effect than the other tissues such as muscle, liver or kidney tissues in fish [7,26]. Accordingly, Feng et al. [27] postulated that only 0.67%–1.60% of ingested inorganic mercury could be converted into methylmercury, which creates the importance of understanding the dietary inorganic mercury toxicity in fish [28]. Adams et al. [9] reported that Hg can deposited naturally in marine fish tissues, in decreasing order, as kidney > liver > muscle > brain > gonad > red blood cells. However, dietary MeHg and inorganic mercury can preferentially bioaccumulate in brain and liver tissues, respectively, using specific bioaccumulation pathway [29]. Cappello et al. [14] reported mercury toxicity in the gills of wild fish using ^1^H NMR-based metabolomics and oxidative stress biomarkers. Furthermore, elevated Hg can cause significant pathological and biochemical changes in marine fish, which may ultimately disturb the health status of fish [9]. Nonetheless, the toxic effect of organic and inorganic mercury may lead to less reproductive success, lower food resource exploitation and predator avoidance problems in fishes, which may threaten their existence in aquatic environment [30].

Olive flounder, *Paralichthys olivaceus* is a commercially important fish species cultured in the East, like Korea, Japan and China [31]. In the republic of Korea, fish consumption, including consumption of olive flounder, has expanded in the last decade. Nevertheless, public health concerns were not properly explored about the consequences of ingesting harmful toxicants. In Korea, fish intake increases up to 50.6 g per individual per day, which comprises at least 3.8% of the total food intake. The amount of organic and total mercury in seafood reaches 1.02 to 780 (mean, 55.6) ng/g wet weight and 4.89 to 1008 (mean, 100) ng/g wet weight, respectively [32]. Since olive flounder, a marine carnivore and demersal fish species, is one of the major food fish in Korea, there is the likelihood of this species being a source of mercury toxicity. Most of the investigations were executed on dietary organic mercury toxicity because inorganic mercury generally is converted to organic mercury along the food chain in nature. Gochfeld [1] reported that dietary fish intake is the most important source of Hg toxicity in humans, of which, around 75%–95% of the mercury can be present in the fish in organic form. However, Ikingura and Akagi [33], and Moon et al. [32] reported that a substantial level of inorganic mercury (0%–44%) can also be present in fish. So far, no research works have reported on toxicity levels of dietary mercury.

Studies on the effects of mercury on the health of fish have been undertaken, however, these studies focused on waterborne exposure to inorganic mercury or dietary exposure to methylmercury, and most studies dealt with field-oriented research [20,34,35,36,37,38,39]. Therefore, as both organic and inorganic mercury are toxic in nature, this study aims to compare the dietary threshold levels for the organic and inorganic mercury toxicity in juvenile olive flounder by using methylmercury (MeHg) and mercuric chloride (HgCl_2_) as dietary organic and inorganic sources of mercury, respectively. The study also compares the tissue level organic or inorganic bioaccumulation, as well as growth performance and blood biochemical characteristics of fish to elucidate the impact of mercury on the health status of juvenile olive flounder. 

## 2. Materials and Methods

### 2.1. Diet Preparation

**Figure 1:**
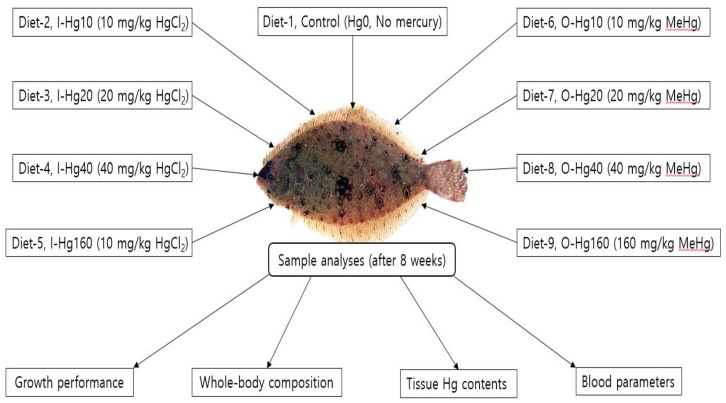
Experimental design and sample analyses of juvenile olive flounder fed with dietary organic (O-Hg) and inorganic (I-Hg) mercury for eight weeks.

Feed formulation and proximate composition of the basal feed is shown in Table 1. Nine semi-purified diets were manufactured in order to contain 50.0% crude protein and 10.0% crude lipid. Four of the diets were formulated to contain 10 (I-Hg10), 20 (I-Hg20), 40 (I-Hg40) or 160 (I-Hg160) mg of inorganic mercury (Hg)/kg diet in the form of mercuric chloride, HgCl_2_ (Sigma-Aldrich, St. Louis, MO, USA); four diets were prepared to contain 10 (O-Hg10), 20 (O-Hg20), 40 (O-Hg40) or 160 (O-Hg160) mg of organic Hg/kg diet in the form of methylmercury, MeHg (Sigma-Aldrich, St. Louis, MO, USA), while one diet was formulated to contain no mercury (Figure 1). We used dietary casein and fish meal as the sources of protein, as well as wheat flour, dextrin, and corn starch as the sources of carbohydrates; and fish oil as the source of lipid. In this study, the inclusion rates of dietary organic and inorganic mercury were selected based on the previously reported dietary experiment on mercury by Lee et al. [39]. 

All the ingredients in dried condition were mixed together with the inclusion of water and oil [31]. Before the pelleting process, Hg was added to 200 mL distilled water, mixing with other feed ingredients. The mixed ingredients became a dough, then the dough was passed through a pelletizer (3 mm diameter) and finally pellets were kept in the open air about 48 h for drying. The dried pellets were then broken and sieved to have the right particle size; the broken pellets were kept in zipper bags, and were stored at −20 °C until feeding of fish.

### 2.2. Fish and Husbandry Settings

All procedures used in the present study were approved by the Animal Ethics Committee Regulations of Pukyong National University, Busan, Rep. of Korea (Protocol number 554). An eight-week experiment was conducted in the facilities of the Feeds and Foods Nutrition Research Center, Pukyong National University, Busan, Rep. of Korea. Before the execution of the experimental trial, fish were procured from a local fish farm and transported to the research center and then adapted to the experimental environment for two weeks. The health status of the fish was checked instantaneously upon arrival. In the adaptation time, fish were exposed to a mercury-deficient feed to reduce the whole-body mercury content, if any. The experiment was executed under a semi-recirculation aquaculture system constituting 27 tanks (each having 30 L water), which received clean seawater (0.8 L per min) from the central reservoir. Water temperature of the experiment was controlled by thermostats (18 ± 2 °C) in the central reservoir. Mechanical aeration was supplied to achieve dissolved oxygen up to required level (6.5 ± 0.5 ppm) and the water salinity was maintained up to 33 ppt. The juvenile olive flounder (average weight 3.1 ± 0.05 g, mean ± SD) were divided randomly into nine groups according to the feeding trial, with 84 fish in each group reared in 27 tanks, each of the tanks containing 28 fish (standard stocking density of fish in 30-L water tanks) in triplicate (9 experimental groups × 3 replicates, 756 fish in total). The feeding protocol followed Moniruzzaman et al. [2] with little modification. Briefly, the experimental fish were fed the diets up to ad libitum at a feeding rate of 2.5%–3.5% of wet body weight. During the experimental period, feeding was twice daily at 8:00 and 18:00 h for eight weeks. To ensure the proper feeding of fish and to minimize the transfer of mercury in each tank, water circulation from the central reservoir was stopped for 30 min daily. We minimized the wastage of feed as well as the leaching of mercury into the water by ensuring slow feeding of fish. Uneaten feeds in the tank were immediately collected after siphoning and the water from central reservoir was recirculated. Fortnightly, total fish weight in each tank was measured using electric balance and fish feed was balanced likewise. 

### 2.3. Experimental Sampling and Analyses

At the end of the feeding trial, fish were refrained from eating for 24 h before sampling started, which helped to empty the intestines of the fish with limited or no feed. Eventually, the total number of fish, as well as total weight of fish, in each aquarium were numbered and weighed to obtain data on growth performance, such as percentage of weight gain (WG), specific growth rate (SGR), feed efficiency (FE), protein efficiency ratio (PER) and survival rate. The sampling procedure was previously described by Moniruzzaman et al. [2]. Briefly, fish were removed from each tank and euthanized in tricaine methane sulfonate solution (MS 222, 0.5 g/L; Argent Chemical Laboratories, Redmount, WA, USA). The euthanized fish were blotted dry on paper towels, weighed and length-measured individually. For tissue collection, gill, liver and kidney tissues from euthanized fish were obtained from four fish per tank randomly at dissection, to measure mercury contents in the respective tissues, with the exception of tanks assigned to O-Hg160-containing diets, where only a few fish survived at the end of the experiment. Mercury contents of diet, tissue, and the fish body were measured spectrophotometrically [40]. The Hg contents in tissues were measured by argon gas assisted Inductively Coupled Plasma Mass Spectrometer, ICP-MS (Perkin-Elmer 3300, Waltham, MA, USA) previously described by Lee et al. [41]. Briefly, weighed samples were put into a 250-mL Kjeldahl flask, and 50 mL of HNO_3_ acid was added to the flask. Then, the flask was heated in a heating mantle until the sample was fully digested. Approximately 5 mL of H_2_O_2_ was added to make sure that the sample was totally digested, and the digested sample was diluted with H_2_O. The concentration of Hg in the diluted digest solution was determined by the method of EPA-6020-A by ICP-MS. To ensure the proper recovery, we used a widely recognized reference sample (DORM-2 dogfish liver; National Research Council, Ottawa, ON, Canada) and blanks during the analyses [41]. Hg values were consistently within the certified ranges (95% recovery level). 

Three euthanized fish from each tank (nine fish per dietary treatment) were collected to analyze carcass composition of fish. We followed the Association of Official Analytical Chemists, AOAC [42] methods to determine the proximate composition of the experimental diets as well as carcass composition of whole body of fish. Moisture contents of fish and feeds were measured by drying the samples at 105 °C in an electric dryer. Ash content was measured by burning the samples at 550 °C in a muffle furnace. Kjeldahl method (N × 6.25) was used to ascertain the crude protein contents of the sample after digestion, distillation and titration process. Crude lipid content was measured by ether extraction method using a soxhlet apparatus 1046 (Tacator AB, Hoganas, Sweden). 

Five euthanized fish from each aquarium (15 fish per treatment) were randomly collected for the analysis of fish blood biochemistry [43]. Fish blood samples were taken from the caudal vein with heparinized plastic syringes. The whole blood was subjected to the centrifugation at 5000× *g* for 10 min and then the supernatant (blood serum) was collected and stored at −70 °C for the measurement of blood bio-chemical attributes such as aspartate transaminase (AST), alanine transaminase (ALT), total protein, and cholesterol. The biochemical characteristics were determined by a blood biochemical analyzer (Fuji DRI-CHEM 3500i, Fuji Photo Film, Ltd., Tokyo, Japan).

### 2.4. Statistical Analysis

All data are expressed as the mean ± standard deviation (SD). Normality and homogeneity of data were confirmed by Shapiro–Wilk and O’Brien tests, respectively. The data were analyzed by one-way analysis of variance (ANOVA) test followed by least significant difference (LSD) test to compare the means [2,41,44] on SAS Version 9.0 (SAS Institute, Cary, NC, USA) software program. Mean differences were considered significant when *p <* 0.05. Broken-line regression models [45] based on percentage of WG data were used to determine the threshold levels of organic and inorganic mercury toxicity for juvenile olive flounder. In case of the organic mercury threshold level, we did not consider the percentage of WG data at O-Hg160 diet because the %WG of fish was very low compared with the other treatments of the organic mercury group, which did not support the concept of the broken-line regression model.

## 3. Results

### 3.1. Growth Performances

Weight gain (WG), specific growth rate (SGR), feed efficiency (FE), protein efficiency ratio (PER) and survival of juvenile olive flounder fed with O-Hg or I-Hg diet are shown in Table 2. At the end of eight weeks of the feeding trial, weight gains (WGs) of fish fed the Hg0, I-Hg10 and O-Hg10 diets were significantly higher than those of fish fed the I-Hg20, I-Hg40, I-Hg160, O-Hg20, O-Hg40 and O-Hg160 diets (*p* < 0.05). Also, WGs of fish fed the I-Hg20 diet were significantly higher than those of fish fed the O-Hg40 and O-Hg160 diets. Furthermore, WG of fish fed the O-Hg40 diet was significantly higher than that of fish fed the O-Hg160 diets. However, there were no significant differences in WG among fish fed the Hg0, I-Hg10 and O-Hg10 diets, between fish fed the I-Hg20 and O-Hg20 diets or between fish fed the I-Hg40 and I-Hg160 diets. Specific growth rates (SGRs) of fish fed the Hg0, I-Hg10 and O-Hg10 diets were significantly higher than those of fish fed the I-Hg40, I-Hg160, O-Hg40 and O-Hg160 diets. Also, SGR of fish fed the O-Hg40 diet was significantly higher than that of fish fed the O-Hg160 diet. However, there were no significant differences in SGR among fish fed the Hg0, I-Hg10, I-Hg20, O-Hg10 and O-Hg20 diets, among fish fed the I-Hg20, I-Hg40 and O-Hg20 diets or among fish fed the I-Hg40, I-Hg160 and O-Hg40 diets. Feed efficiency (FE) of fish fed the Hg0, I-Hg10 and O-Hg10 diets was significantly higher than that of fish fed the I-Hg160, O-Hg40 and O-Hg160 diets. Also, FE of fish fed the I-Hg160 diet was significantly higher than that of fish fed the O-Hg160 diet. However, there were no significant differences in FE among fish fed the Hg0, I-Hg10, I-Hg20, I-Hg40, O-Hg10 and O-Hg20 diets, among fish fed the I-Hg20, I-Hg40, O-Hg20 and O-Hg40 diets or among fish fed the I-Hg160 and O-Hg40 diets. Protein efficiency ratio (PER) of fish fed the O-Hg10 diet was significantly higher than that of fish fed the I-Hg160, O-Hg20, O-Hg40 and O-Hg160 diets. Also, PER of fish fed the I-Hg160 diet was significantly higher than that of fish fed the O-Hg160 diet. However, there were no significant differences in PER among fish fed the Hg0, I-Hg10, I-Hg20, I-Hg40 and O-Hg10 diets; among fish fed the Hg0, I-Hg10, I-Hg20, I-Hg40 and O-Hg20 diets; among fish fed the I-Hg20, I-Hg40, O-Hg20 and O-Hg40 diets or among fish fed the I-Hg160 and O-Hg40 diets. There were no significant differences in survival rates of fish in all the experimental diets, with the exception of fish fed the O-Hg160 diet.

### 3.2. Proximate Composition

Proximate composition of the whole-body of juvenile olive flounder fed the diets containing various levels of organic and inorganic mercury for eight weeks is summarized in Table 3. There were no significant differences in whole-body moisture, crude protein, and ash contents among fish fed the experimental diets. However, in the case of whole-body lipid contents, fish fed the control diet showed significantly higher lipid content than that of fish fed the O-Hg40 diet. There were no clear trends to show the increasing or decreasing pattern in terms of whole-body moisture, crude protein and crude lipid contents among fish fed the experimental diets, excluding the O-Hg160-containing diet, since most of the fish died at the experimental period.

### 3.3. Mercury Concentration

Total bioaccumulations of mercury in the liver, kidney and gill tissues of fish fed the experimental diets for eight weeks are shown in Table 4. Whole-body Hg burden increased as the dietary inclusion level of Hg increased. Mercury concentrations in liver, kidney and gill tissues of fish fed the O-Hg40 diets were significantly higher than those of fish fed the diets containing Hg0, I-Hg10, I-Hg20, I-Hg40, I-Hg160, O-Hg10 and O-Hg20 diets (*p* < 0.05). No significant differences were found in total mercury concentrations in liver, kidney and gill tissue of fish fed the Hg0, I-Hg10 and I-Hg20 diets (*p* ≥ 0.05). Meanwhile, the kidney showed the highest MeHg concentration, followed by liver and gill. On the other hand, liver tissue bioaccumulated the highest HgCl_2_ concentration, followed by kidney and gill tissues. 

### 3.4. Blood Biochemical Parameters

Blood biochemical characteristics of juvenile olive flounder fed diets having various levels of organic and inorganic mercury are shown in Table 5. No significant differences were observed in total protein, AST, ALT and cholesterol contents of fish fed the experimental diets.

### 3.5. Broken Line Analysis

**Figure 2:**
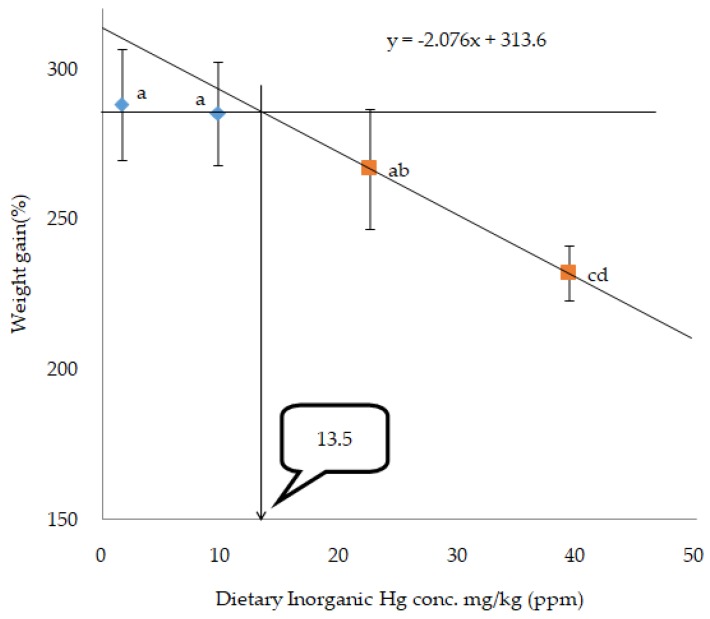
Broken-line analysis of weight gain (%) in olive flounder fed different levels of dietary inorganic mercury (HgCl_2_) for eight weeks_._

**Figure 3:**
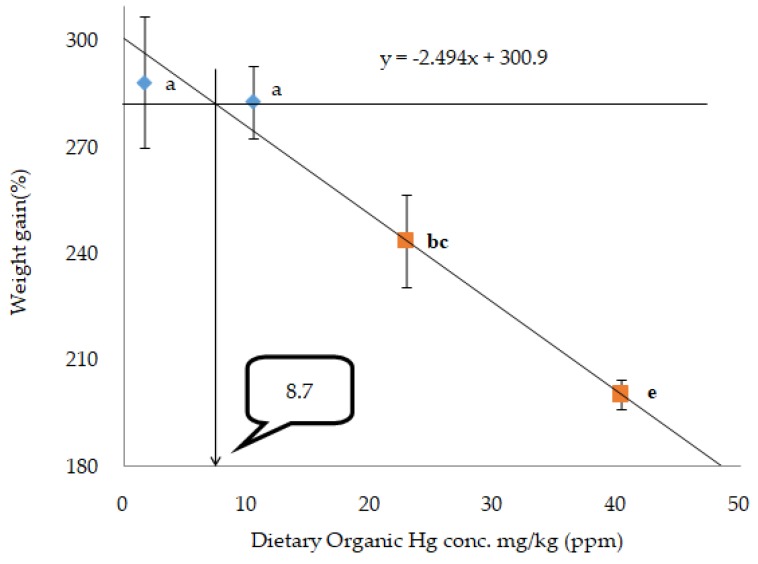
Broken-line analysis of weight gain (%) in olive flounder fed different levels of dietary organic mercury (MeHg) for eight weeks.

The broken-line regression model for percentage of WG indicated that the dietary Hg threshold toxicity level could be 13.5mg Hg/kg in the form of HgCl_2_ (Figure 2) and 8.7 mg Hg/kg (Figure 3) in the form of MeHg in juvenile olive flounder.

## 4. Discussions

In the present study, organic and inorganic mercury concentrations ranging from 10 (O-Hg and I-Hg) mg to 160 (O-Hg and I-Hg) mg/kg diets were tested. Fish fed diets containing (O-Hg160) mg/kg showed the highest toxic effect compared with those fed diets containing less organic, and irrespective of inorganic mercury concentrations, all fish died, except a few fish (no fish in one tank) at termination of the experiment, which were also not adequate for further sample analyses. In this study, the high mortality of fish indicates that oral administration of organic mercury is more toxic in juvenile olive flounder than its counterpart inorganic mercury. In previous studies on teleost fishes, Rodgers and Beamish [46] postulated that rainbow trout showed no toxic effect when they were fed 25, 45, or 95 mg MeHg/kg diets in an experiment that lasted for 12 weeks. In an another research work, Houck and Cech [47] revealed that Sacramento blackfish did not show any significant effect on their mortality when fed 22.2 or 55.5 mg MeHg/kg diets in a research work for 10 weeks. However, when Sacramento blackfish were fed diets with 55.5 mg MeHg/kg, after 35 weeks a remarkable mortality of fish was observed, which means experimental duration is an important factor to determine the toxicity level in fish. Furthermore, dietary MeHg exhibited a higher toxic effect in juvenile beluga sturgeon (*Huso huso*) than green sturgeon; all fish died when the beluga sturgeon were fed a 16.22-mg MeHg/kg diet in a feeding trial for six weeks [39,48], which means toxicity in fish may also be determined by type of fish species. So, the studies confirmed that mercury toxicity may vary from fish species to species and duration of the toxicity exposure time. In the present study, the broken-line regression model showed that the threshold level for Hg toxicity might be 13.5 mg Hg/kg when it is incorporated in the feed in the form of HgCl_2_, and 8.7 mg Hg/kg in the form of MeHg, respectively, based on the percentage WG of fish. In agreement with the present study, Niimi and Kisson [13] reported that 10–20 mg of Hg/kg could be toxic to fish. Some studies have reported the speculation of a diet incorporating optimum mercury toxicity levels, which might be toxic to the fish body. Wobeser [49] observed that dietary mercury as a 24 mg/kg diet did not affect mortality in rainbow trout (*Oncorhynchus mykiss*). The results also suggested that trout can tolerate a large body burden of mercury (30 mg/kg diet) if this mercury is acquired over a period of time. On the contrary, Friedmann et al. [50] found decreased growth performance in juvenile walleye (*Stizostedion vitreum*) when fish were fed fish muscle containing mercury at 0.1 mg/kg wet weight. The main route of mercury poisoning in animals can occur by easy enchaining of Hg^2+^ (mercuric ion) to the thiol (glutathione, thioredoxins and glutaredoxins) or sulfhydryl (SH) groups of proteins (cysteine) [2,41]. The enchaining with hydroxyl, carboxyl, and phosphoric groups may also lead to mercury toxicity [3]. SH-groups play the key role in architectural and functioning properties of many proteins, however, they may cause a falling off of protein functions, damage the structure of proteins, as well as disorder in the protein operation system, if they are enchained with mercury ion [51]. 

The proximate composition of the whole-body of fish fed various concentrations of organic and inorganic mercury for eight weeks showed no obvious effects on moisture, crude protein, and ash contents of fish fed the experimental diets. Also there was no trend to show in terms of carcass content analyses of fish fed the diets. Nevertheless, the carcass contents of fish fed the O-Hg40mg/kg diet showed higher ash, but lower crude lipid. Previous study indicated that the MeHg-incorporated diet had no remarkable effects on carcass composition in terms of moisture, crude protein, lipid, or energy contents of white and green sturgeon [39]. Nonetheless, the moisture content was significantly higher in green sturgeon than in white sturgeon according to the experimental trial, on the other hand, carcass composition of the fish body in terms of crude protein, lipid, and energy concentrations in white sturgeon was more noteworthy than that in green sturgeon in all feeding trials [39]. In the present study, whole-body lipid content was significantly lower in fish fed the O-Hg40 diet than the control diet, which might be due to expending more energy for repairing damaged cells within allocated dietary energy on high toxicity in fish [47]. 

According to Van der Oost et al. [52], bioaccumulation should be addressed, including toxicokinetics, metabolism, and organ-specific bioaccumulation. In this study, fish fed the O-Hg40 mg/kg diets showed significantly higher Hg concentrations in liver, kidney, and gill tissues than fish fed the reduced amount of methylmercury and mercuric chloride-incorporated diets. The result might be due to the rapid methylation process, high bioavailability and low excretion of organic mercury in the tissue levels [1]. For the time being, the kidney showed the highest organic Hg concentration followed by liver and gill tissues (kidney > liver > gills). In the case of inorganic mercury, a higher level of Hg content was found in liver tissue, followed by kidney and gill tissues (liver > kidney > gills). In agreement with our findings, Gentes et al. [29] reported that dietary inorganic mercury can highly bioaccumulate in liver tissue of fish with the exhibition of toxicity. In addition, the researchers reported that organic mercury can also be highly deposited in brain and liver tissue of fish [29], which also supported the present findings. In the present study, increased mercury load in the experimental diets resulted in a great accumulation in fish tissues, which is in agreement with Rowland et al. [53], who opined that manufacturing of feeds affects the bioaccumulation of mercury and the harmful effects in organisms. Moreover, a higher level of dietary organic mercury has been found to accumulate in the gill, liver and kidney tissues, compared with dietary inorganic mercury in the fish, which agrees with the results reported by Simon and Boudou [54] in carp, *Ctenopharyngodon idella*. 

Hematological parameters are mostly used as the potential indicators for the assessment of fish health status [43]. Blood serological characteristics of juvenile olive flounder fed various concentrations of organic and inorganic mercury-incorporated diets showed insignificant effects on some metabolites such as total protein, AST, ALT and cholesterol from fish in all experimental diets. Interestingly, in the present study, AST and ALT levels were not affected by dietary organic or inorganic mercury levels, even though growth performance of fish was affected. The result might be attributed to the higher level of Hg deposition and the detoxifying capacity of the fish liver as the largest digestive gland. However, because of the variations in the strength and duration of the serological responses and lack of validated and standardized procedures, detection of fish antibodies has not yet been accepted as a routine diagnostic method for assessing mercury toxicity level in fish. During the experimental period, no external lesions have been found in fish except abnormal movement and moribund fish, which in turn died. 

## 5. Conclusions

In conclusion, according to the broken-line regression model for percentage of WG, the threshold/optimum toxicity level of a mercury-incorporated diet for juvenile olive flounder could be 13.5 mg Hg/kg in terms of HgCl_2_ and 8.7 mg Hg/kg in terms of MeHg, respectively. This study has novel implications for further study on the mechanisms of toxicity and its detoxification by different antioxidants at different temperatures in the marine fish model. 

## Figures and Tables

**Table 1:** Feed formulation and proximate composition of the basal diet (% of dry matter, DM basis).

Ingredients	% In Diet
Casein ^1^	27.0
Fish meal ^2^	32.0
Wheat flour ^3^	18.0
Dextrin ^1^	4.20
Corn starch ^2^	3.50
Fish oil ^4^	6.10
Vitamin premix ^5^	3.00
Mineral premix ^6^	3.00
Cellulose ^1^	3.20
Hg-premix (5000 ppm)	0.00

^1^ Sigma Aldrich 63103, St. Louis, MO, USA. ^2^ Suhyup Feed Co. Ltd., Uiryeong, Republic of Korea. ^3^ Young Nam Flour Mills Co., Busan, Republic of Korea. ^4^ Ewha Oil Co. Ltd., Busan, Republic of Korea. ^5^ contains (as mg/kg in diets): Ascorbic acid, 300: Inositol, 150; Menadione, 6; Niacin, 150; Pyridoxine. HCl, 15; Riboflavin, 30; Thiamine mononitrate, 15; dl-a-Tocopherol acetate, 201; Retinyl acetate, 6; Biotin, 1.5; B12, 0.06. ^6^ contains(as mg/kg in diets): NaCl, 437; MgSO_4_.7H_2_O, 1379.8; ZnSO_4_.7H_2_O, 226.4; Fe-Citrate, 299; MnSO_4_, 0.016; FeSO_4_, 0.378; CuSo4, 0.00033; Calcium iodate, 0.0006; MgO, 0.00135; NaSeO_3_, 0.00025.

**Table 2:** Growth performances of juvenile olive flounder, *Paralichthys olivaceus,* fed nine experimental diets for eight weeks ^1.^

Diets	WG (%) ^2^	SGR (%/day) ^3^	FE (%) ^4^	PER ^5^	SR (%) ^6^
Hg0	288.4 ± 18.5 ^a^	2.77 ± 0.10 ^a^	109.8 ± 10.6 ^a^	2.33 ± 0.22 ^a,b^	81.7 ± 7.6 ^a,b^
I-Hg10	285.3 ± 17.1 ^a^	2.75 ± 0.09 ^a^	106.4 ± 6.6 ^a^	2.29 ± 0.14 ^a,b^	83.3 ± 5.8 ^a,b^
I-Hg20	266.9 ± 19.9 ^b^	2.65 ± 0.11 ^a,b^	102.1 ± 7.2 ^a,b^	2.16 ± 0.15 ^a,b,c^	85.0 ± 5.0 ^a,b^
I-Hg40	232.1 ± 9.06 ^c,d^	2.45 ± 0.1 ^b,c,d^	95.8 ± 11.7 ^a,b^	2.06 ± 0.25 ^a,b,c^	78.3 ± 2.8 ^a,b^
I-Hg160	207.6 ± 6.33 ^d,e^	2.29 ± 0.1 ^c,d^	77.4 ± 3.8 ^c^	1.65 ± 0.08 ^d^	75.0 ± 5.0 ^a,b^
O-Hg10	282.9 ± 10.2 ^a^	2.74 ± 0.05 ^a^	110.2 ± 7.9 ^a^	2.34 ± 0.17 ^a^	81.7 ± 2.8 ^a,b^
O-Hg20	243.8 ± 12.9 ^b,c^	2.52 ± 0.08 ^a,b,c^	94.4 ± 6.2 ^a,b^	1.99 ± 0.13 ^b,c^	86.7 ± 5.8 ^a^
O-Hg40	200.4 ± 3.9 ^e^	2.24 ± 0.03 ^d^	87.0 ± 13.9 ^b,c^	1.87 ± 0.3 ^c,d^	73.3 ± 14.4 ^b^
O-Hg160	7.3 ± 20.8 ^f^	0.12 ± 0.38 ^e^	4.1 ± 11.1 ^d^	0.07 ± 0.25 ^e^	13.3 ± 10.4 ^c^

^1^ Values are mean from triplicate groups (*n* = 3) of flounder where the values in each column with different superscripts are significantly different (*p* < 0.05). ^2^ WG (%) = (final weight-initial weight) × 100/initial weight. ^3^ SGR (%/day) = (loge final weight – loge initial weight) × 100/day ^4^ FE (%) = (wet weight gain (g) × 100/dry feed intake) ^5^ PER = Wet weight gain (g)/Protein intake (g) ^6^ SR (%): (total stocked fish-dead fish at the end) × 100/total stocked fish. ^a–f^ Data with different superscripts are significantly different.

**Table 3:** Whole-body proximate composition of juvenile olive flounder, *Paralichthys olivaceus,* fed nine experimental diets for eight weeks ^1^.

Diets	Moisture (%)	Crude Protein (%)	Crude Lipid (%)	Ash (%)
Hg0	76.3 ± 0.8	70.5 ± 1.1	11.7 ± 1.4 ^a^	12.7 ± 1.2
I-Hg10	77.7 ± 0.8	70.2 ± 1.6	11.1 ± 2.1 ^a,b^	13.3 ± 0.7
I-Hg20	77.5 ± 0.4	69.4 ± 2.2	11.2 ± 1.2 ^a,b^	14.1 ± 4.5
I-Hg40	78.3 ± 1.0	69.1 ± 1.4	10.7 ± 0.42 ^a,b^	14.1 ± 1.5
I-Hg160	77.7 ± 0.9	69.0 ± 2.2	9.9 ± 2.5 ^a,b^	14.4 ± 0.7
O-Hg10	77.8 ± 1.8	70.0 ± 1.4	11.0 ± 1.4 ^a,b^	13.4 ± 1.4
O-Hg20	77.5 ± 1.0	69.7 ± 1.4	9.1 ± 0.7 ^a,b^	14.2 ± 1.6
O-Hg40	77.3 ± 3.0	69.1 ± 1.6	8.8 ± 1.3 ^b^	15.2 ± 0.7
O-Hg160	ND	ND	ND	ND

ND = not detectable due to inadequate sample ^1^ Values are mean from triplicate groups (*n* = 3) of flounder where the values in each column with different superscripts are significantly different (*p* < 0.05). ^a,b^ Data with different superscripts are significantly different.

**Table 4:** Tissue mercury concentrations (μg/g of wet matter basis) of juvenile olive flounder, *Paralichthys olivaceus,* fed experimental diets for eight weeks ^1^.

Diets	Gill (μg/g)	Kidney (μg/g)	Liver (μg/g)
Hg0	1.83 ± 0.51 ^d^	2.02 ± 0.96 ^e^	1.38 ± 0.31 ^e^
I-Hg10	1.63 ± 0.37 ^d^	2.30 ± 0.31 ^e^	1.52 ± 0.37 ^e^
I-Hg20	1.77 ± 0.17 ^d^	2.88 ± 0.51 ^e^	3.12 ± 0.64 ^d,e^
I-Hg40	2.55 ± 0.29 ^d^	5.09 ± 0.95 ^d,e^	7.55 ± 5.13 ^c^
I-Hg160	3.37 ± 0.30 ^c,d^	13.8 ± 1.79 ^c^	17.1 ± 2.18 ^b^
O-Hg10	5.32 ± 0.87 ^b,c^	8.44 ± 0.56 ^d^	6.69 ± 1.68 ^c,d^
O-Hg20	7.58 ± 1.32 ^b^	17.74 ± 2.18 ^b^	14.53 ± 2.39 ^b^
O-Hg40	13.82 ± 3.64 ^a^	28.29 ± 4.59 ^a^	24.20 ± 2.53 ^a^
O-Hg160	ND	ND	ND

ND = not detectable due to inadequate sample ^1^ Values are mean from triplicate groups (*n* = 3) of flounder where the values in each column with different superscripts are significantly different (*p* < 0.05). ^a–e^ Data with different superscripts are significantly different.

**Table 5:** Blood biochemical characteristics of juvenile olive flounder, *Paralichthys olivaceus,* fed nine experimental diets for eight weeks ^1^.

Diets	Total Protein ^2^	AST ^3^	ALT ^4^	Cholesterol ^5^
Hg0	1.8 ± 0.42	242 ± 59.6	15 ± 8.72	141 ± 23.4
I-Hg10	2.2 ± 0.36	297 ± 52.1	11.7 ± 0.58	160 ± 13.1
I-Hg20	2.2 ± 0.25	224 ± 49.24	9.0 ± 1.73	155 ± 0.0
I-Hg40	2.0 ± 0.38	268 ± 52.1	12.3 ± 1.53	150 ± 19.8
I-Hg160	1.7 ± 0.40	225 ± 38.4	10.7 ± 2.52	140 ± 13.4
O-Hg10	1.9 ± 0.10	224 ± 51.9	11.0 ± 2.65	167 ± 4.51
O-Hg20	1.7 ± 0.40	254 ± 121.9	11.7 ± 6.66	142 ± 27.5
O-Hg40	1.7 ± 0.35	232 ± 48.8	11.3 ± 3.21	152 ± 14.5
O-Hg160	ND	ND	ND	ND

ND = not detectable due to inadequate sample ^1^ Values are mean from triplicate groups (*n* = 3) of flounder where the values in each column with different superscripts are significantly different (*p* < 0.05). ^2^ Total protein (mg/dL) ^3^ AST: Aspartate transaminase ^4^ ALT: Alanine transaminase ^5^ Cholesterol (mg/dL).
